# Cross-sectional MRI-based quantification of central nervous system tissue and cerebrospinal fluid volumes in juvenile domestic pigs

**DOI:** 10.3389/fvets.2026.1861994

**Published:** 2026-07-08

**Authors:** Luke S. Myers, John F. Griffin, Sarah G. Christian, Scott V. Dindot

**Affiliations:** 1 Department of Veterinary Pathobiology, College of Veterinary Medicine & Biomedical Sciences, Texas A&M University, College Station, TX, United States; 2 Department of Large Animal Clinical Sciences, College of Veterinary Medicine & Biomedical Sciences, Texas A&M University, College Station, TX, United States; 3 Translational Imaging Center (TIC), College of Veterinary Medicine & Biomedical Sciences, Texas A&M University, College Station, TX, United States; 4 Ultragenyx Pharmaceutical Inc., Novato, CA, United States

**Keywords:** CNS measurements, CNS volume, CSF volume, MRI, pig

## Abstract

**Introduction:**

There is a growing need for alternative animal models to evaluate central nervous system (CNS)-targeted therapies, and pigs are emerging as a promising option. Their translational utility depends on accurate estimates of CNS tissue and cerebrospinal fluid (CSF) volumes, which are important for the design and interpretation of preclinical dosing studies.

**Methods:**

A cross-sectional magnetic resonance imaging (MRI) study was conducted in 12 domestic Yorkshire–Landrace pigs (*Sus scrofa*) across four postnatal age groups (2, 5, 11, and 19 weeks). MRI was performed on the brain and spinal cord using T2-weighted turbo spin echo with fat saturation and short *tau* inversion recovery sequences. CNS tissue and CSF volumes were segmented and quantified for brain and spinal compartments, along with linear measurements of brain cavity dimensions, spinal cord and canal lengths, and regional spinal cord and subarachnoid space dimensions.

**Results:**

Age-associated differences were observed across groups, including increases in CNS tissue and CSF volumes, CNS length, and multiple brain and spinal anatomical measurements. Notably, spinal CSF volume exceeded brain CSF volume in older pigs, indicating a shift in CSF compartmental distribution that may be relevant to dosing and delivery considerations for CNS-targeted therapies.

**Conclusion:**

This study provides MRI-derived, age-specific estimates of brain and spinal CSF and CNS tissue volumes in juvenile pigs, which may inform anatomical assumptions and study design considerations for CNS-focused preclinical research.

## 1 Introduction

Preclinical research on central nervous system (CNS) therapies primarily relies on rodent and non-human primate (NHP) models (1–4); however, both models have notable limitations. Rodents, while readily accessible, often exhibit poor translatability to humans due to their small size, substantial anatomical differences, and limited predictive value for pharmacokinetic (PK), pharmacodynamic (PD), and toxicological outcomes (5–7). NHPs are anatomically and physiologically more similar to humans, yet their use is increasingly constrained by ethical considerations, high costs, limited availability, and the lack of genetically modified disease models (8).

These challenges have created a growing need for alternative preclinical models, particularly large animal models. Domestic pigs (*Sus scrofa*) are an attractive option due to their anatomical and physiological similarities to humans, cost-effectiveness, and ease of maintenance (9). They can produce large litters in a relatively short period, and there is an expanding repertoire of genetically engineered pig models for CNS disorders, including Huntington’s disease, Batten disease, and Angelman syndrome (7, 9–11). However, to effectively leverage pigs in CNS research, a deeper understanding of their CNS tissue and cerebrospinal fluid (CSF) volumes would greatly strengthen their utility in these applications.

Accurate CNS and CSF volume estimates support the design of preclinical PK/PD and toxicology studies and inform dose selection considerations in translational research (5). This is particularly important in studies involving CNS-targeted therapies, such as gene therapies, antisense oligonucleotides, and biologics, which are typically administered through intracerebroventricular, cisterna magna, or lumbar intrathecal routes (5, 6, 12).

The combined CSF and CNS tissue volumes in pigs have not been previously quantified. To address this gap, we conducted a cross-sectional magnetic resonance imaging (MRI) study using T2-weighted turbo spin echo (TSE) with fat saturation and short *tau* inversion recovery (STIR) scans of the brain and spinal cord in 12 pigs. Four age groups were examined (*n* = 3 per group): 2 weeks (3.9 kg), 5 weeks (7.5 kg), 11 weeks (28.7 kg), and 19 weeks (53.3 kg). We quantified CSF and CNS tissue volumes, along with linear measurements of brain cavity dimensions, spinal cord and canal lengths, and regional spinal cord and subarachnoid space dimensions along the cervical, thoracic, and lumbar spine.

## 2 Methods

### 2.1 Animals and housing

This study was approved by the Institutional Animal Care and Use Committee (IACUC) at Texas A&M University, approval number 2023–0179. Sample size was constrained by project funding and breeding availability. Twelve Yorkshire–Landrace pigs (5 females, 7 intact males) were generated via artificial insemination and selected from 10 independent litters derived from 2 boars and 8 sows (Supplementary Table 1) to maximize genetic variability given the small cohort size. Pigs remained with their sows until weaning at 21 days of age. Animals were assigned to four postnatal age groups as follows: 2 weeks (14 days; *n* = 3, 1F/2M), 5 weeks (30–37 days; *n* = 3, 2F/1M), 11 weeks (73–80 days; *n* = 3, 1F/2M), and 19 weeks (134–137 days; *n* = 3, 1F/2M; Supplementary Table 1).

Pigs were group-housed in climate-controlled rooms (74 ± 1 °F [23.3 ± 0.6 °C], 12-h light/dark cycles, 40–60% humidity). Animals were fed age-appropriate commercial diets, and feed was provided ad libitum until 40 days of age (M-G Pig Starter 20% - Gen 2.0, Welmar, TX, USA) and was subsequently adjusted to 4 and 2% of body weight daily for pigs aged 40–120 days and >120 days (M-G Hog Pellets 14%, Welmar, TX, USA), respectively. Animals were weighed the day prior to MRI acquisition and underwent a veterinary physical examination on the morning of imaging to confirm they were clinically healthy. Feed was withheld overnight (approximately 12 h) prior to anesthesia, while water remained available ad libitum.

### 2.2 Anesthesia and positioning

Anesthesia was induced with an intramuscular injection of Telazol (3–5 mg/kg; Zoetis Inc., MI, USA) to permit endotracheal intubation. Following intubation, pigs were maintained under inhalant anesthesia using 1 to 3% isoflurane, adjusted individually as needed (Aspen Veterinary Resources, MO, USA), and mechanically ventilated with a Vetland Landmark VSA-2100 Anesthesia System. To minimize motion artifacts and ensure consistent positioning, animals were placed in the supine position. Body temperature was maintained with heating mats and blankets and continuously monitored using a rectal temperature probe. Blood pressure (cuff) and oxygen saturation (pulse oximetry) were monitored throughout imaging.

### 2.3 Magnetic resonance imaging protocol

All MRI scans were performed using a 3 T scanner (70 cm wide-bore, Magnetom Verio, Siemens Healthineers, Erlangen, Germany) to obtain images of the brain and spinal cord. Two sequences were acquired for each animal: a T2-weighted TSE sequence with fat saturation and a STIR sequence. The T2-weighted TSE sequence was acquired in the sagittal plane using the adaptive inline-compose feature, producing a composite image that included the brain and entire spinal column. The STIR sequence, obtained in the transverse plane, provided fat suppression to enhance visualization of CSF and other fluid-containing structures. Both sequences used inline composition to ensure consistent image contrast and complete anatomical coverage across the full spinal length.

Scan times ranged from 1.5 to 2 h depending on the number of acquisition blocks required for complete spinal composition. The number of acquisition blocks was related to subject body size. Pigs were positioned in the supine position within the scanner, and images were reoriented for publication to standard anatomical orientation. The brain was positioned within a Siemens neck coil using the eyes and ears as anatomical landmarks to ensure adequate brain coverage. Siemens spine and dual body matrix (flex) coils were used concurrently to optimize signal reception and maintain complete coverage of the spinal axis. Throughout the procedure, radiofrequency pulse settings were standardized to low specific absorption rate to ensure safety and image uniformity.

### 2.4 Sequence parameters

For the sagittal T2-weighted turbo spin echo (TSE) sequence, imaging parameters included a repetition time (TR) of 4,800 ms, an echo time (TE) of 87 ms, a field of view (FoV) of 225 mm with 100 percent phase coverage, a matrix size of 320 × 80, and a slice thickness of 2 mm. The flip angle was 120 degrees, and two signal averages were acquired.

The transverse short *tau* inversion recovery (STIR) sequence was acquired with a TR of 8,830 ms, a TE of 32 ms, an FoV of 125 mm with 100 percent phase coverage, a matrix size of 256 × 100, and a slice thickness of 3 mm. The flip angle was 120 degrees, and generalized autocalibrating partially parallel acquisition (GRAPPA) was enabled with an acceleration factor of 2.

### 2.5 CSF and tissue segmentation

Segmentation and volumetric measurements were performed using 3D Slicer software (version 5.10.0) (13). The paint and auto-fill tools were used to manually segment the ventricles and subarachnoid space in the STIR transverse images, which provided strong contrast between CSF and surrounding soft tissues. Volumetric values were calculated from voxel dimensions multiplied by the segmented voxel counts. No thresholding or post-processing steps were applied following manual segmentation. For Pig ID 10, T2-weighted sagittal images were used because the STIR acquisition was affected by a technical issue that prevented adequate image quality.

Window and level settings were adjusted locally across the image series to optimize visualization of CSF boundaries and surrounding tissues for manual segmentation; these adjustments did not alter underlying voxel intensities. Brain CSF was defined as the lateral, third, and fourth ventricles and the cranial subarachnoid space. Spinal CSF was defined as the subarachnoid space extending from the foramen magnum to the lumbar cistern. Combined CSF and combined tissue volumes were calculated as the sum of the respective brain and spinal measurements, with the foramen magnum serving as the anatomical boundary between compartments. Volume-to-body weight ratios (cm^3^/kg) were calculated by dividing combined CSF and combined CNS tissue volumes by individual body weight.

All segmentations were performed by a single trained investigator using standardized anatomical landmarks and consistent segmentation criteria to ensure uniform application across animals. Segmentation was performed in two stages: CSF spaces were delineated first, followed by segmentation of all CNS tissue. The two segmentations were overlaid, and each slice was reviewed to confirm that no voxels were assigned to both compartments and that no regions were unclassified. This quality-control step ensured complete and non-overlapping delineation of CSF and tissue throughout the full dataset.

### 2.6 CNS measurements

CNS dimensions were assessed using the line and curve tools in 3D Slicer (version 5.10.0) (13). Measurements included brain cavity length, height, and width; spinal cord and spinal canal lengths; and spinal cord and canal heights at key vertebral levels. Lengths and heights were measured on T2-weighted sagittal images with fat saturation.

Spinal cord length was measured from the foramen magnum to the caudal extent of the conus medullaris, which is long and tapered in pigs and typically terminates near the second sacral vertebra (S2) (14). Spinal canal length was measured from the foramen magnum to the caudal aspect of the lumbar cistern. Brain cavity length was defined as the maximal distance from the cribriform plate to the occipital bone. Brain cavity height was measured on the anatomical mid-sagittal plane, identified in 3D Slicer, as the perpendicular distance at the level of the Sella turcica. Brain cavity width was determined on STIR transverse images at the widest point of the slice containing the rostral colliculi.

Spinal cord height was measured at the first cervical vertebra (C1), the first thoracic vertebra (T1), and the first lumbar vertebra (L1), and spinal canal height was recorded at the widest point of each corresponding vertebra, which were adequately visualized on the T2-weighted sagittal sequence. These vertebral levels were selected as representative landmarks within the cervical, thoracic, and lumbar regions, allowing assessment of regional variation in spinal cord and subarachnoid space dimensions along the neuraxis. These regions also correspond to anatomical areas commonly considered during cisterna magna access and lumbar intrathecal delivery procedures in large-animal studies. Spinal subarachnoid space height was calculated as (spinal canal height – spinal cord height)/2. CNS length was measured from the center of the eye to the first identifiable sacral vertebra on MRI, excluding Pig ID 11 due to imaging limitations. This measure was preferred over snout-to-tail length because of variability in snout length and tail docking practices.

### 2.7 Statistics

Statistical comparisons were only made between two independent age groups (2-week-old vs. 19-week-old pigs; *n* = 3 animals per group) using two-tailed Welch’s *t*-tests to account for unequal variances. Given the small sample size (*n* = 3 per group), formal tests of normality were not performed. Analyses were conducted in JMP Pro 18 (SAS Institute Inc., Cary, NC, USA), and results are reported as t(_df_) with corresponding *p*-values. Statistical significance was set at *α* = 0.05.

Given the cross-sectional design and limited sample size within each age group, statistical analyses were intended to identify group-level differences across the sampled age range rather than to provide definitive inference regarding developmental trajectories. Sex was not included as a variable in statistical models due to limited statistical power.

## 3 Results

### 3.1 Cross-sectional differences in body weight, CNS length, and CSF and CNS tissue volumes across postnatal age groups

**Figure 1:**
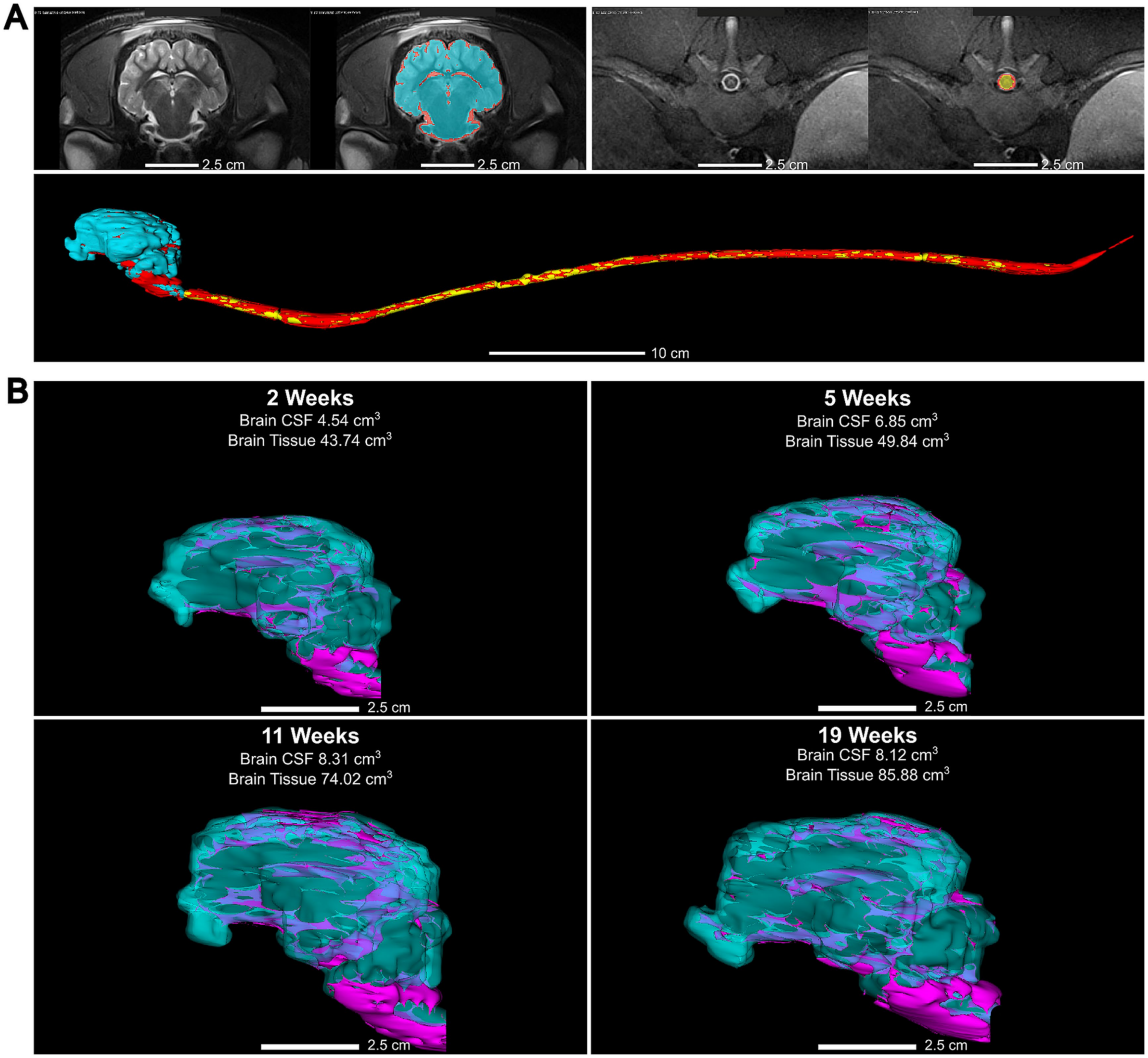
MRI-based segmentation workflow and age-associated differences in brain CSF and CNS tissue volumes. **(A)** Representative segmentation workflow from an 11-week-old pig (ID 8). Left panels show transverse STIR MRI slices of the brain and spinal cord prior to segmentation. Adjacent panels show manual segmentation of CSF (red) and CNS tissue (brain: cyan; spinal cord: yellow). The bottom panel shows a three-dimensional rendering of the segmented CSF and CNS tissue spanning the brain and entire spinal cord from the same animal. **(B)** Representative three-dimensional renderings of segmented brain CSF (purple) and brain tissue (cyan) from individual pigs at 2, 5, 11, and 19 weeks of age. Values shown correspond to individual animals and are provided for illustrative comparison of age-associated differences in brain CSF and tissue volumes. Segmentation and rendering were performed using 3D Slicer (13). CSF, cerebrospinal fluid; CNS, central nervous system. *Accessible description*: Figure shows MRI-based segmentation of cerebrospinal fluid (CSF) and central nervous system (CNS) tissue in pigs. Panel **(A)** presents transverse MRI slices of the brain and spinal cord before and after manual segmentation, with CSF highlighted in red, brain tissue in cyan, and spinal cord tissue in yellow. A three-dimensional rendering illustrates segmented CSF and CNS tissue spanning the entire brain and spinal cord in an 11-week-old pig. Panel **(B)** displays three-dimensional renderings of segmented brain CSF (purple) and brain tissue (cyan) from representative pigs at 2, 5, 11, and 19 weeks of age, showing visible increases in overall brain size and CSF volume with age.

To determine CSF volumes and CNS tissue volumes across postnatal age groups, a cross-sectional MRI study was conducted in 12 Yorkshire–Landrace pigs distributed across four age groups (Table 1): 2 weeks (*n* = 3), 5 weeks (*n* = 3), 11 weeks (*n* = 3), and 19 weeks (*n* = 3). All MRI datasets were suitable for quantitative analysis. Brain CSF measurements included the lateral, third, and fourth ventricles as well as the cranial subarachnoid space (Figure 1A). Spinal CSF encompassed the subarachnoid space extending from the foramen magnum to the lumbar cistern (Figure 1A). The foramen magnum served as the anatomical boundary between brain and spinal compartments, and combined CSF and combined CNS tissue volumes represent the sum of the respective brain and spinal measurements.

**Table 1:** Body weight, CNS length, and CSF and CNS tissue volumes.

Measurement	2 weeks			5 weeks			11 weeks			19 weeks		
	Pig 1	Pig 2	Pig 3	Pig 4	Pig 5	Pig 6	Pig 7	Pig 8	Pig 9	Pig 10	Pig 11	Pig 12
											
Sex	M	F	M	F	M	F	M	M	F	F	M	M
Body weight (kg)	3.7	3.8	4.3	7.8	7.2	7.3	26.0	33.0	27.0	57.0	47.0	56.0
CNS length (cm)	37.4	37.2	38.3	46.4	47.2	48.1	68.8	74.1	72.5	86.3	N/A	87.7
Combined CNS tissue (cm^3^)	49.5	49.4	48.1	56.9	58.6	67.8	95.9	88.5	94.4	115.5	115.0	120.5
Combined CNS tissue/body weight (cm^3^/kg)	13.4	13.1	11.3	7.3	8.1	9.3	3.7	2.7	3.5	2.0	2.4	2.2
Brain tissue (cm^3^)	42.9	43.7	42.2	48.4	49.8	56.8	75.3	69.7	74.0	84.5	85.9	89.1
Spine tissue (cm^3^)	6.6	5.7	5.9	8.6	8.8	11.0	20.6	18.7	20.4	31.0	29.1	31.3
Combined CSF (cm^3^)	8.0	7.7	8.0	10.7	11.8	12.3	19.9	19.3	19.7	23.6	25.3	23.2
Brain CSF (cm^3^)	4.6	4.5	4.7	5.6	6.8	6.6	9.4	8.2	8.3	8.1	8.1	8.2
Spine CSF (cm^3^)	3.4	3.1	3.3	5.1	5.0	5.6	10.5	11.1	11.4	15.5	17.2	15.0
Combined CSF/body weight (cm^3^/kg)	2.2	2.0	1.9	1.4	1.6	1.7	0.8	0.6	0.7	0.4	0.5	0.4

Combined CNS tissue represents the sum of brain and spinal cord tissue volumes. M, male; F, female; CSF, cerebrospinal fluid; CNS, central nervous system; N/A, data not available.

Across the 2- to 19-week age range, body weight increased substantially, from an average of 3.9 kg at 2 weeks to 53.3 kg at 19 weeks (T_(2)_ = 15.5, *p* = 0.004), representing an approximately 13-fold increase (Table 1; Supplementary Table 1). CNS length increased approximately 2.3-fold, from 37.6 cm in 2-week-old pigs to 87.0 cm in 19-week-old pigs (T_(1.5)_ = 65.3, *p* = 0.0015; Table 1; Supplementary Table 1). A similar pattern was observed for combined CNS tissue volume, which increased approximately 2.4-fold, from 49 cm^3^ to 117 cm^3^ (T_(2.2)_ = 37.4, *p* = 0.0003; Table 1; Figure 1B; Supplementary Table 1). Combined CSF volume exhibited a somewhat greater relative increase, rising approximately 3-fold from 7.9 cm^3^ (1 cm^3^ = 1 mL) in 2-week-old pigs to 24.1 cm^3^ in 19-week-old pigs (T_(2.1)_ = 24.6, *p* = 0.0012; Table 1; Figure 1B; Supplementary Table 1). When expressed relative to body weight (cm^3^/kg), combined CNS tissue and combined CSF values were lower in the 19-week-old group compared to the 2-week-old group (Table 1).

A pronounced age-associated difference was observed in the relative contribution of spinal and brain CSF volumes. In 2-week-old pigs, spinal CSF volume represented approximately 70% of brain CSF volume, whereas in 19-week-old pigs, the spinal CSF volume represented 195% of the brain CSF volume (Table 2). Additional age-specific comparisons of CNS tissue and CSF volumes are summarized in Table 2, with the largest proportional differences observed in the spinal CSF compartment.

**Table 2:** Proportional relationships between CNS and CSF volumes across pig age groups.

Ratio of CNS and CSF measurements (%)	2 weeks	5 weeks	11 weeks	19 weeks
Spine CSF/brain CSF	71.0 ± 2.6	83.1 ± 8.9	128.1 ± 14.3	195.5 ± 15.1
Spine tissue/brain tissue	14.1 ± 1.2	18.3 ± 1.0	27.3 ± 0.4	35.2 ± 1.4
Brain CSF/brain tissue	10.7 ± 0.4	12.3 ± 1.2	11.8 ± 0.6	9.4 ± 0.2
Spine CSF/spine tissue	53.9 ± 2.2	55.7 ± 4.3	55.4 ± 4.2	52.3 ± 5.9
Combined CSF/combined CNS tissue	16.1 ± 0.5	19.0 ± 1.0	21.1 ± 0.6	20.6 ± 1.4

Values represent mean ± SD ratios expressed as percentages, calculated from individual animal measurements within each age group. CS, cerebrospinal fluid; CNS, central nervous system.

### 3.2 Cross-sectional differences in brain cavity and spinal cord and canal anatomy across postnatal age groups

**Figure 2:**
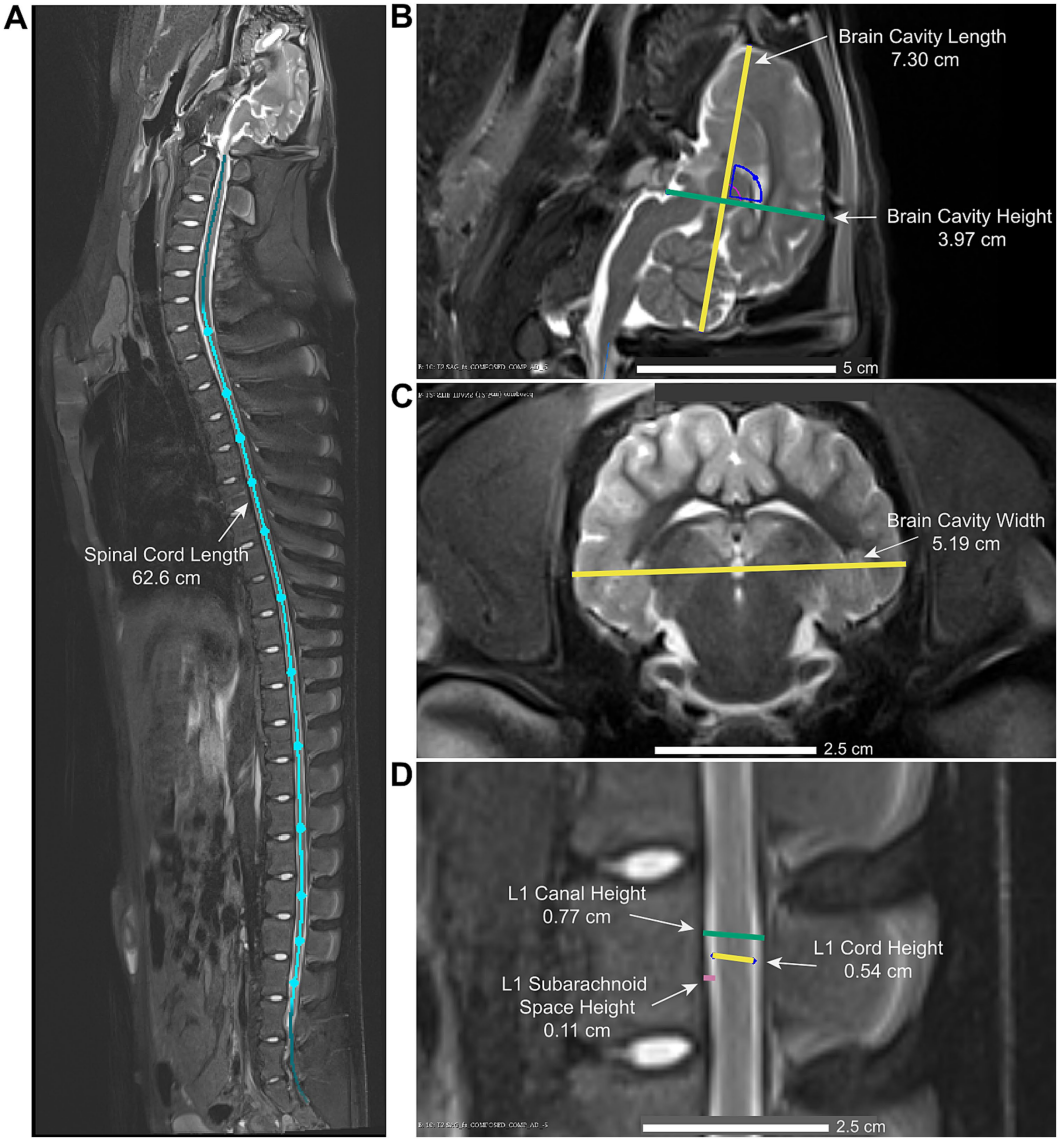
Representative CNS measurement approaches in pigs. Representative MRI images illustrating CNS measurement methods in a representative pig (ID 8) from the 11-week-old age group. Panels **(A)**, **(B)**, and **(D)** show T2-weighted turbo spin echo (TSE) with fat saturation sagittal images, and panel **(C)** shows a transverse short *tau* inversion recovery (STIR) image. **(A)** Spinal cord length measured from the foramen magnum to the caudal extent of the conus medullaris. **(B)** Brain cavity dimensions, including maximal brain cavity length measured from the cribriform plate to the occipital bone and brain cavity height measured orthogonally at the level of the Sella turcica. **(C)** Brain cavity width measured at the widest point of the transverse slice containing the rostral colliculi. **(D)** L1 spinal cord height, spinal canal height, and subarachnoid space height measured at the L1 vertebral level. Measurements were performed using 3D Slicer (13). L1, First Lumbar Vertebra (L1). *Accessible description*: The figure contains four MRI panels demonstrating how CNS measurements were obtained in a representative 11-week-old pig. Panel **(A)** shows a sagittal T2-weighted MRI image with a line indicating spinal cord length measured from the foramen magnum to the caudal extent of the conus medullaris. Panel **(B)** shows a sagittal MRI image of the brain with lines indicating brain cavity length and brain cavity height measured at the level of the sella turcica. Panel **(C)** shows a transverse STIR MRI image with a line indicating brain cavity width measured at its widest point. Panel **(D)** shows a sagittal MRI image of the lumbar spine with lines indicating spinal cord height, spinal canal height, and subarachnoid space height at the L1 vertebral level. Measurement locations are labeled directly on each image.

Spinal cord length increased approximately 2.3-fold, from 30.9 cm in 2-week-old pigs to 69.6 cm in 19-week-old pigs (T_(2.1)_ = 16.5, *p* = 0.0028; Table 3; Supplementary Table 1), with representative measurement locations shown in Figure 2A. In contrast, changes in brain dimensions were more modest. Brain length increased approximately 1.3-fold, from 6.1 cm to 7.8 cm (T_(3.5)_ = 13.5, *p* = 0.0004), while brain width increased approximately 1.2-fold, from 4.6 cm to 5.6 cm (T_(3.5)_ = 12, *p* = 0.0005) over the same age range (Figures 2B,C; Table 3; Supplementary Table 1).

**Table 3:** Anatomical CNS measurements in 2, 5, 11 and 19-week-old pigs.

Measurement (cm)	2 weeks			5 weeks			11 weeks			19 weeks		
	Pig 1	Pig 2	Pig 3	Pig 4	Pig 5	Pig 6	Pig 7	Pig 8	Pig 9	Pig 10	Pig 11	Pig 12
Spinal cord length	30.6	30.4	31.7	38.4	38.8	39.6	56.6	62.6	59.3	71.1	65.1	72.7
Spinal canal length	32.8	32.7	33.7	41.9	40.4	44.0	61.9	64.6	64.7	79.4	N/A	77.6
Brain cavity length	6.0	6.1	6.2	6.1	6.4	6.5	7.4	7.3	7.2	7.7	7.7	8.0
Brain cavity width	4.6	4.7	4.5	4.8	4.7	5.0	5.4	5.2	5.4	5.5	5.6	5.7
Brain cavity height	3.4	3.3	3.3	3.5	3.5	3.5	3.8	4.0	3.8	4.1	4.2	4.3
C1 spinal cord height	0.46	0.50	0.54	0.55	0.55	0.63	0.68	0.53	0.66	0.68	0.71	0.82
T1 spinal cord height	0.49	0.48	0.50	0.52	0.56	0.64	0.72	0.60	0.71	0.66	0.75	0.76
L1 spinal cord height	0.33	0.39	0.41	0.43	0.50	0.47	0.50	0.54	0.58	0.63	0.71	0.69
C1 canal height	0.74	0.73	0.75	0.83	0.82	0.91	0.93	0.80	1.02	1.08	0.94	1.07
T1 canal height	0.67	0.72	0.73	0.85	0.80	0.99	1.01	0.88	1.03	0.89	1.14	1.09
L1 canal height	0.56	0.57	0.57	0.66	0.65	0.65	0.78	0.77	0.80	0.85	0.99	0.96
C1 SAS height	0.14	0.12	0.10	0.14	0.14	0.14	0.13	0.13	0.18	0.20	0.11	0.12
T1 SAS height	0.09	0.12	0.11	0.17	0.12	0.18	0.14	0.14	0.16	0.12	0.19	0.17
L1 SAS height	0.11	0.09	0.08	0.12	0.08	0.09	0.14	0.11	0.11	0.11	0.14	0.14

N/A, data not available; C1, first cervical vertebra; T1, first thoracic vertebra (T1); L1, first lumbar vertebra; SAS, subarachnoid space.

Consistent patterns were observed across other anatomical measurements, with the largest proportional increases occurring in the spinal region (Table 3; Supplementary Table 1). Measurements of spinal cord and subarachnoid space height were obtained at the C1, T1, and L1 vertebral levels, with representative measurement locations illustrated in Figure 2D. At C1, mean spinal cord height increased approximately 1.5-fold, from 0.50 cm in 2-week-old pigs to 0.74 cm in 19-week-old pigs (T_(3.1)_ = 5.1, *p* = 0.014; Table 3; Supplementary Table 1). At L1, spinal cord height increased approximately 1.8-fold, from 0.38 cm to 0.68 cm (T_(4)_ = 9.2, *p* = 0.0008) over the same interval (Table 3; Supplementary Table 1). In contrast, subarachnoid space height exhibited smaller proportional changes. At C1, subarachnoid space height increased from 0.12 cm to 0.15 cm (T_(2.7)_ = 0.77, *p* = 0.5; approximately 1.2-fold), while at L1 it increased from 0.10 cm to 0.13 cm (T_(4)_ = 2.41, *p* = 0.074; approximately 1.4-fold; Table 3; Supplementary Table 1).

## 4 Discussion

This study provides a cross-sectional characterization of CSF volumes, CNS tissue volumes, and major anatomical measurements in domestic pigs between 2 and 19 weeks of age. MRI-based segmentation of brain and spinal compartments demonstrated age-associated differences in both tissue and CSF volumes, with the most substantial changes observed in the spinal region. These data present practical reference values for investigators using pigs in neuroimaging, developmental studies, or preclinical modeling.

Across the sampled ages, brain cavity measurements increased modestly, whereas spinal cord and canal dimensions showed larger proportional changes. One notable observation was the shift in relative compartmental CSF distribution: spinal CSF volume was lower than brain CSF volume at younger ages but exceeded brain CSF volume in older animals. This pattern is consistent with the larger proportional expansion of the spinal compartment relative to the cranial compartment across the sampled age range. Although the study design does not allow evaluation of individual trajectories, the cross-sectional trends highlight that the balance between cranial and spinal CSF compartments is not static during juvenile growth. Developmental changes in CNS anatomy and CSF volumes have been reported in humans and other species, indicating that CNS compartment proportions are dynamic throughout growth (15, 16).

These age-associated differences in CSF distribution and CNS length may influence the dilution, distribution, and transport of therapeutics administered through CNS-targeted delivery routes. Consequently, exposure patterns following lumbar intrathecal, cisterna magna, or intracerebroventricular administration may vary across developmental stages. Studies involving intrathecal dosing or anatomical modeling may benefit from incorporating age-specific compartmental volumes rather than relying on a single, pooled CSF estimate. Similarly, the observed changes in volume-to-body-weight ratios indicate that CSF and CNS tissue volumes do not scale proportionally with body weight across development. These findings suggest that body-weight-based dose scaling alone may not adequately account for age-dependent differences in CNS anatomy when designing CNS-targeted therapeutic studies.

Pigs occupy an intermediate position between commonly used rodent models and larger translational species such as non-human primates and humans with respect to CNS size and anatomical complexity (17). Consequently, they may provide a useful platform for evaluating CNS-targeted therapeutics in situations where compartment dimensions, CSF distribution, and transport distance are expected to influence drug exposure. Although direct quantitative comparisons across species are complicated by differences in age, methodology, and anatomical definitions, the present findings highlight that CSF compartmental distribution and CNS dimensions should not be assumed to scale proportionally across developmental stages or species. These data therefore provide a reference framework that may assist investigators when interpreting CNS delivery studies and designing translational dosing strategies.

Several limitations should be considered when interpreting these findings. The small sample size within each age group (*n* = 3) limited statistical power and the precision of variance estimates, while the cross-sectional study design restricted interpretation to differences between age groups rather than within-animal developmental changes; therefore, statistical comparisons should be interpreted cautiously and in the context of the consistent directional trends observed across related anatomical measurements. All animals were derived from a single commercial Yorkshire–Landrace population, and anatomical proportions may differ across breeds, genetic lines, or management systems.

Additional limitations relate to the imaging and segmentation methodology. All segmentations and anatomical measurements were performed by a single trained investigator, and formal intra-rater and inter-rater reproducibility assessments were not conducted. Although standardized segmentation criteria were applied across all animals and CSF and CNS tissue segmentations were reviewed on a slice-by-slice basis to confirm complete, non-overlapping compartment delineation, manual segmentation remains susceptible to operator-dependent variability, image contrast, windowing, partial-volume effects, and anatomical boundary definitions. In addition, volumetric estimates relied exclusively on MRI-based segmentation because independent cadaveric validation was not feasible. Measurements derived from T2-weighted and STIR MRI sequences may also be influenced by the high signal intensity of CSF and finite spatial resolution, and thin regions of the cranial subarachnoid space may not have been fully captured during segmentation. Future studies incorporating higher-resolution imaging protocols, alternative MRI sequences, or complementary imaging modalities may further improve anatomical accuracy. Consequently, the reported dimensions of CSF-containing structures and associated volumetric estimates should be interpreted as anatomical approximations rather than exact physical measurements.

## 5 Conclusion

In summary, this study provides MRI-derived CSF and CNS tissue measurements across key juvenile stages in domestic pigs. These data offer a reference framework for selecting age-appropriate anatomical assumptions in porcine research and may assist in designing studies that require accurate estimates of cranial and spinal CSF compartment volumes.

## Data Availability

The raw data supporting the conclusions of this article will be made available by the authors, without undue reservation.
